# Moxibustion of Zusanli (ST36) and Shenshu (BL23) Alleviates Cartilage Degradation through RANKL/OPG Signaling in a Rabbit Model of Rheumatoid Arthritis

**DOI:** 10.1155/2019/6436420

**Published:** 2019-01-03

**Authors:** Yang Chen, Haijun Li, Xiaochao Luo, Huahui Liu, Yumei Zhong, Xiao Wu, Xuguang Liu

**Affiliations:** ^1^Acupuncture and Tuina School, Chengdu University of Traditional Chinese Medicine, Chengdu 610075, China; ^2^People's Hospital of Qingbaijiang District of Chengdu City, Chengdu 610300, China; ^3^Chinese Medicine Hospital Affiliated to Southwest Medical University, Luzhou 646000, China

## Abstract

Rheumatoid arthritis (RA) is a systemic and chronic autoimmune inflammatory disease characterized by severe synovial hyperplasia associated with progressive cartilage degradation. Due to the severe pain and disability caused by RA, effective therapeutic strategies that could simultaneously alleviate the inflammatory response and delay the disease progression are urgently needed. As a major alternative therapy in traditional Chinese medicine, moxibustion has been demonstrated that it could reduce the chronic inflammatory responses of a series of musculoskeletal diseases; however, whether moxibustion has protective effects on RA is still unclear. To investigate the effects of moxibustion on RA, moxibustion was applied to Zusanli (ST36) and Shenshu (BL23) acupoints in a RA rabbit model. HE staining of articular cartilage showed that moxibustion alleviated the cartilage degradation and bone destruction. In addition, moxibustion decreased the osteoclast number in RA rabbits. Real-time PCR revealed that moxibustion decreased the expression of RANKL mRNA while increased the expression of OPG mRNA, indicating a restoration of the balance between osteogenesis and osteoclastogenesis. Taken together, our results indicated that moxibustion had promising antiarthritic effects and could be an useful alternative method in RA therapeutics.

## 1. Introduction

Rheumatoid arthritis (RA) is a chronic, systemic, and complicated autoimmune osteopathic disease. Its enigmatic pathogenesis is characterized by persistent synovitis and progressive cartilage degradation and bone erosion, leading to severe disability over time [1]. RA is most common in females and the estimated prevalence is 0.5-1% in adult population [2]. Although the etiology of RA is currently not fully elucidated, a combination of genetic and environmental risk factors has been proposed to play an important role in RA pathogenesis [3]. The goal of RA treatment is to remission or reduce disease activity, ultimately slowing or preventing the progression of joint destruction.

Bone erosion is the most prominent feature of RA and is highly correlated with disease progression and functional outcome. Bone erosion results from the disturbed balance between bone resorption and bone formation, which has been arbitrated upon the initiation of osteoclastogenesis by activating osteoclast [4]. Considerably, the osteoclast has become widely recognized as the cell responsible for bone erosions and is also required for articular bone resorption in RA [5]. Receptor activator of nuclear factor B ligand (RANKL) has been proved as a major protagonist in activation of osteoclast and is the most essential factor for osteoclastogenesis [6]. RANKL stimulates the osteoclast precursor cells differentiating into osteoclasts [7] and other pleiotropic cytokines (TNF-a, IL-17, IL-6, and IL1-*β*) expedite this process, either by incorporation with RANKL or inducing directly [8]. Inflamed synovial tissues provide several cellular sources of RANKL, including lymphocytes and fibroblast-like synoviocytes [9]. Moreover, accumulated evidences have suggested that RANKL level is positively correlated with bone erosion in RA [10]. Of note, osteoprotegerin (OPG), the decoy receptor of RANKL, acts as the natural inhibitor of RANKL and prevents RANKL from binding to its osteoclast receptor [11]. OPG influences bone resorption negatively and OPG knockout mice exhibit severe osteoporosis and bone erosion [12], implicating the importance of RANKL/OPG balance for maintaining osteoclast homeostasis. Thus, downregulation of these transcription factors potentially attenuates the proinflammatory mediator's production, which has been proved essential for maintaining the physiological bone homoeostasis in RA [8].

Current therapeutics against RA, either anti-inflammatory or biological agents, have side effects (cardiovascular and gastrointestinal lineages complications) or develop into drug resistance [13, 14]. On to this premise, the perspective of this study was to explore other therapeutic method that could disparage the pathogenesis of RA. Moxibustion, a traditional Chinese medicine treatment, is commonly used in clinics as a combination of pharmacological material and nonpharmacological practices. Previous studies have demonstrated that moxibustion reduces chronic inflammatory response in several musculoskeletal disease states, including knee osteoarthritis [15], cervical spondylosis [16], and primary osteoporosis [17]. These studies link moxibustion to remolding the bone metabolism in inflammatory diseases; however, the questions remain whether moxibustion alleviates the cartilage damage in RA.

In the present study, we used histological staining and real-time polymerase chain reaction (real time PCR) to observe the effects of moxibustion on cartilage degradation and mRNA expression of RANKL and OPG in the knee joints of RA rabbits, to discover the protective effects and putative underlying mechanism of moxibustion on RA.

## 2. Materials and Methods

### 2.1. Animals

Thirty skeletally mature, male (15) or female (15), Japanese white rabbits (weight, 2.5 ± 0.25 kg) were obtained from Experimental Animal Center of Chengdu University of Traditional Chinese Medicine and were acclimated for one week prior to the experimental procedures. All animals were allowed free access to food and water ad libitum in controlled room temperature (23 ± 1°C) with 12-hour light-dark cycles. All animal procedures were approved by the Institutional Ethics Committee.

### 2.2. Establishment of RA in Rabbits

After one week of acclimation, rabbits were randomly divided into 3 groups (n = 10 for each group): control group, RA group, and RA with moxibustion group. A rabbit model of RA was established by injection of Freund's Complete Adjuvant (FCA; Sigma-Aldrich, St. Louis, MO) into the stifle knee joints, as described in previous study [18]. FCA contains heat-killed dead mycobacterium tuberculosis bacteria in liquid paraffin in the concentration of 10 mg/ml. All the rabbits, except those in the control group, were injected with a single dose of FCA (0.25 ml/kg) in the triangular region formed by the patellar tendon, lower edge of the patella, and tibial plateau of the stifle knee joints. All the animals developed the signs of arthritis such as swelling, redness, and restricted movement of the knee. Rabbits in the control group received the same volume of saline.

### 2.3. Moxibustion Method

**Figure 1:**
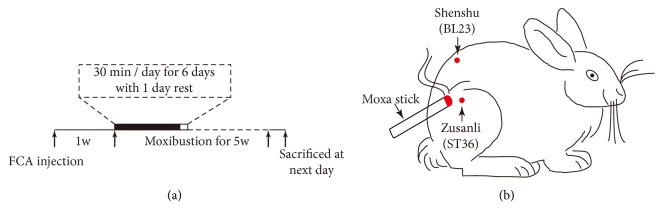
**Experimental illustration**. (a) Time line of experiment. (b) Schematic diagram showing that rabbit received moxibustion at acupoints of Zusanli (ST 36) and Shenshu (BL23).

Moxibustion treatment was performed 7 days after FCA injection (Figure 1(a)). In the present study, the location of acupoints was determined based on the stands of the Experimental Acupuncture [19]. Zusanli (ST36) was located at the upper one-fifth point of dorsolateral of leg and Shenshu (BL23) was located at the second lumbar spinous process adjacent to open 1.5 cm (Figure 1(b)). The rabbits were fixed to the experimental table and fur on the skin was shaved to expose the acupoints. Animals-used moxa sticks (diameter: 8 mm, length: 20 cm, Nanyang Dongsheng Moxibustion Technology Development Co., Ltd., China) were burned to carry out moxibustion 2-3 cm over the acupoints for avoiding skin burned, which may be related with stress response. The moxibustion treatments were performed for 30 min per day for consecutive 5 weeks and there was 1-day rest at the seventh day of each week (Figure 1(a)). Rabbits in the control group were restricted for 30 min without moxibustion treatment.

### 2.4. Immunohistochemical Experiments

Rabbits were sacrificed at the next day after the last trial of moxibustion treatment (Figure 1(a)). Rabbits were anesthetized with 25% urethane (4 ml/kg; Shanghai-Rui Biological Technology Co., Ltd., Shanghai, China) and sacrificed by air injection (10 ml) into ear marginal vein. The knee joints of right rear leg were incised and the quadriceps were dissected 0.5 cm above the knee joint. A size of 0.5 cm × 0.5 cm × 0.3 cm articular bone containing the cartilage was removed using bone ribbing rongeur. The samples were rinsed in ice-cold phosphate-buffered saline solution (PBS; Zhongshanjinqiao Biological Technology Co., Ltd., Beijing, China) and fixed in 10% neutral buffered formalin (Chengdu Kelong Chemical Reagent Factory, China) for 24 h. The samples were then decalcified in 10% ethylene diamine tetraacetic acid (EDTA; Sigma-Aldrich, St. Louis, MO) and embedded in paraffin wax for cartilage degradation. Hematoxylin-eosin (HE) staining was used to assess cartilage surface characteristic, cells (hypercellularity or cell colony formation), hypocellularity, and empty lacunae. For HE staining, the embedded cartilages were sectioned in a microtome at 5 *μ*m and stained with HE (Bailingwei Technology Co., Ltd., Beijing, China). For immunohistochemistry, sections were dewaxed and submerged in 3% hydrogen peroxide for 10 min. Sections were then blocked with normal goat serum at room temperature for 30 min and incubated with rabbit anti-RANKL (1:200, Bioss, Woburn, MA, USA) in blocking solution at 4°C overnight. Afterward, sections were washed in PBS for 3 times and incubated with biotinylated secondary antibody at 37°C for 30 min, followed by signal amplification with streptavidin biotin-horseradish peroxidase (37°C, 30 min) and staining with diaminobenzidine (DAB). At last, sections were conterstained with hematoxylin.

Images were acquired using inverted microscope equipped with digital cameras (Olympus photomicroscope, Tokyo, Japan). Histological analysis was conducted by another experimenter who was blinded to the experimental manipulation in each of the three groups. Cartilage degradation was scored according to the Mankin score [20], which assigns scores ranging from 0 for normal cartilage to 5 for the most severe damage. For the evaluation of articular structure, 0 to 5 refer to normal structure, irregular cracks on the surface, cracks reaching transitional layer, cracks reaching radiation layer, and cracks reaching calcified layer and abscission of cartilage layer, respectively. For the evaluation of cartilage cells, 0 to 3 refer to normal number of cartilage cells, diffuse increase of cartilage cells, appearance of large number of cell clusters, and obviously decreased cell number, respectively. For the evaluation of tidemark integrity, 0 to 2 refer to normal tidemark, multiple tidemark, and subchondral blood vessels invading the tidemark, respectively. For evaluation of the effects of moxibustion on osteoclast, osteoclasts were characterized as RANKL^−^ cells and the number of osteoclast was counted.

### 2.5. Real-Time PCR Experiment

Cartilage samples were collected from each group as described above. The RNA extraction was performed according to the method described previously [18]. Briefly, the samples were combined with the Trizol reagent (Thermo Fisher Scientific, Inc., Waltham, MA, USA) according to the manufacturer's instructions. Total RNA was extracted and 2 *μ*g mRNA was used to transcribe cDNA using RevertAid First Strand cDNA Synthesis kit (Thermo Fisher Scientific Inc., Waltham, MA, USA). mRNA expression of *β*-actin and the selected molecules were determined by using SYBR Green Master Mix (Applied Biosystems, Foster City, CA, USA). Primer sequence used were as follows: *β*-actin, sense 5′-GAAGATCAAGATCATTGCTCCT- 3′ and antisense 5′ -TACTCCTGCTTGCTGATCCA- 3′; RANKL, sense 5′-TTTGCAGGACTCGACTCTGGAG- 3′ and antisense 5′ -TCCCTCCTTTCAGGTTATGAG- 3′; OPG, sense 5′-ATCATTGAATGGACAACCCAGG- 3′ and antisense 5′ -TGCGTGGCTTCTCTGTTTCC- 3′. The PCR cycling reaction was performed as follows: denaturation at 95°C for 30 s, followed by 40 PCR cycles of denaturation at 95°C for 5 s, annealing at 55°C for 30 s, and extension at 72°C for 30 s; subsequently, samples were held at 4°C until use. Data were collected and analyzed using Sequence Detection Software (Applied Biosystems, CA, USA). The relative expression of target gene was calculated with the threshold cycle value (CT) for each sample compared with the endogenous *β*-actin control. The relative expression level of each gene was presented as fold change and quantified using 2^−ΔΔCT^ method.

### 2.6. Statistical Analysis

Data were expressed as the mean ± SD. Results were analyzed by SPSS 19.0 statistical software. Difference between groups was evaluated by one way ANOVA test followed by LSD post hoc comparison. Kruskal-Wallis test followed by post hoc Mann-Whitney U test was used when there was lack of homogeneity of variances. P < 0.05 was considered statistically significant.

## 3. Results

### 3.1. Moxibustion Treatment Attenuates RA

**Figure 2:**
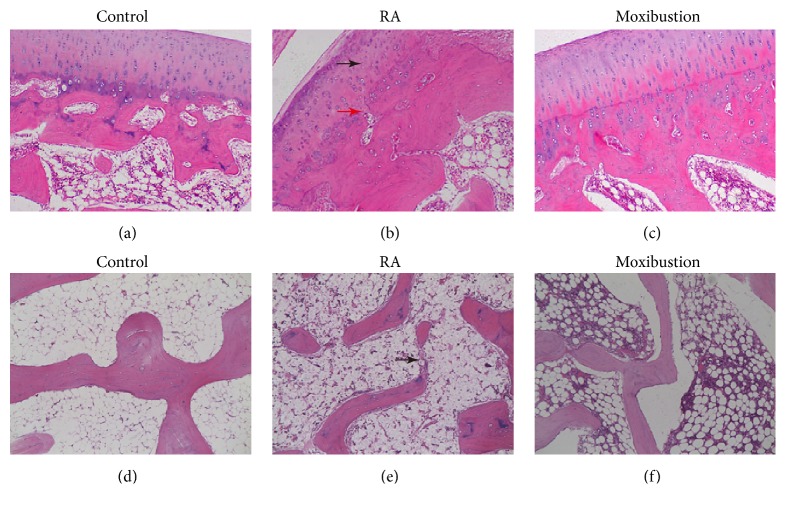
**Histological assessment cartilage degradation in control, RA, and moxibustion group**. ((a) to (c)) HE staining showing the changes of articular structure, cartilage cells and tidemark integrity in control (a), RA (b), and moxibustion group (c). Black arrow indicates disordered chondrocyte. Red arrow indicates destruction of tidemark. ((d) to (f)) HE staining showing the changes of subchondral bone trabeculae in control (d), RA (e), and moxibustion group (f). Black arrow indicates disruption of bone trabecular. Magnification, × 400.

A rabbit model of RA was established by injection of FCA into the knee, as described in previous study [18]. Following FCA treatment, the pathological changes of articular bone in the knees were assessed using HE staining. The alterations of cartilage structure, cartilage cells, and tidemark integrity were analyzed. In the control group, there was no pathological evidence of cartilage degeneration. The cartilage showed smooth surface, arranged chondrocyte, and integral tidemark (Figure 2(a)). The subchondral bone trabeculae in control group were orderly arranged and there was no disruption (Figure 2(d)). In contrast, the RA group displayed typical pathological changes cartilage degeneration, including partial damage of cartilage surface, disordered arrangement of chondrocyte, tidemark destruction, and a thickening of cartilage layer (Figure 2(b)). In addition, there were decreased area of subchondral bone trabeculae, increased empty lacunae, and destruction of subchondral bone trabeculae (Figure 2(e)).

To investigate whether moxibustion has protective effects on RA, we performed moxibustion on Zusanli (ST36) and Shenshu (BL23) in RA rabbits (Figure 1(b)). 5 weeks after moxibustion, we assessed the pathological changes of cartilage using HE staining. HE staining revealed that pathological changes of cartilage were attenuated after moxibustion treatment. Compared with RA group, the level of cartilage surface damage, chondrocyte arrangement, and tidemark integrity were significantly improved (Figure 2(c)). Moreover, there was less destruction of subchondral bone trabeculae in moxibustion group compared with RA group (Figure 2(f)).

**Figure 3:**
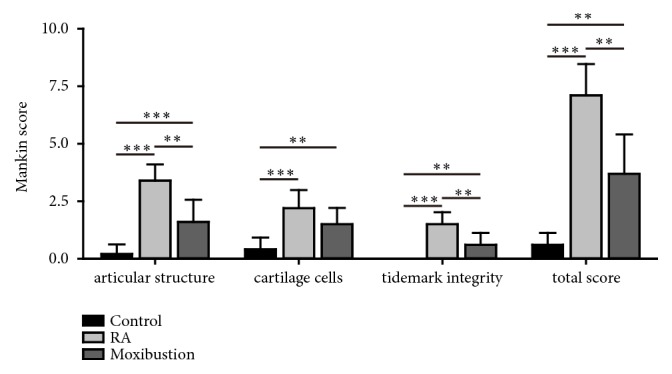
**Effects of moxibustion on Mankin scores**. The articular structure, cartilage cells, and tidemark integrity were analyzed in rabbits in control, RA, and moxibustion group. Histopathological grading of the cartilage was assessed using the Mankin score system. n =10 for each group, the Kruskal-Wallis test followed by post hoc Mann-Whitney U test. *∗∗* P < 0.01; *∗∗∗* P < 0.001. Data were expressed as the mean ± SD.

Cartilage changes were evaluated according to the Mankin score system. The Mankin scores of articular structure, cartilage cells, and tidemark integrity were assessed, respectively. Compared with control group, Mankin score was significantly increased in RA group (Figure 3, RA group: cartilage structure: 3.4 ± 0.69; cartilage cells: 2.2 ± 0.79; tidemark integrity: 1.5 ± 0.53; total score: 7.1 ± 1.37). However, the moxibustion group showed significantly lower Mankin scores than that in RA group (Figure 3, moxibustion group: cartilage structure: 1.6 ± 0.97; cartilage cells: 1.5 ± 0.71; tidemark integrity: 0.6 ± 0.52; total score: 3.7 ± 1.70). These results suggested that moxibustion treatment attenuated the pathological changes of RA.

### 3.2. Moxibustion Treatment Inhibits the Osteoclastogenesis Process in RA

**Figure 4:**
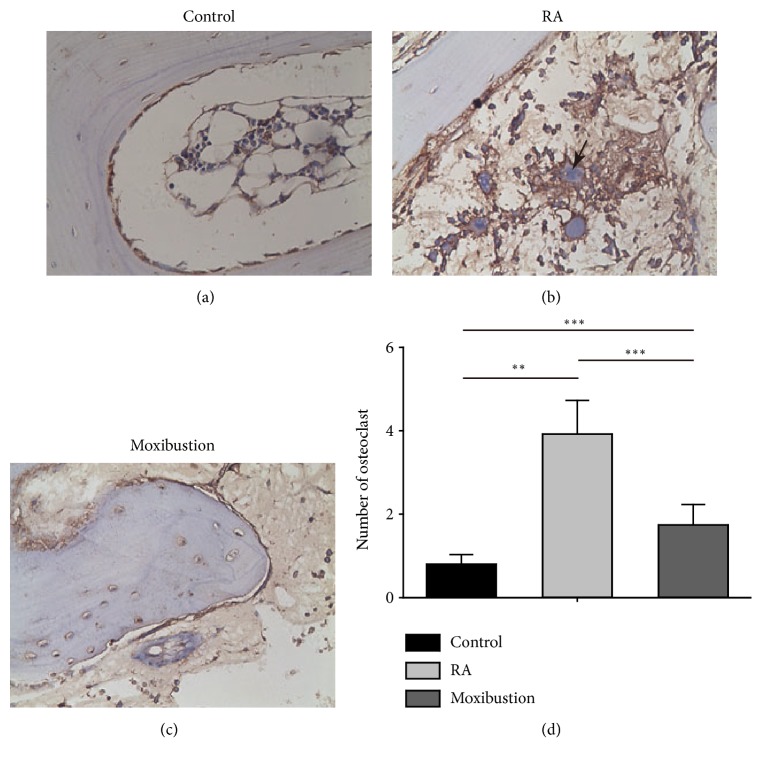
**Histological assessment the number of osteoclast in control, RA, and moxibustion group**. ((a) to (c)) Representative images showing the expression of osteoclast in control (a), RA (b), and moxibustion (c) group. Black arrow indicates the position of an RANKL negative osteoclast cell. Magnification, × 400. (d) Statistical analysis of the number of osteoclast. n =10 for each group, the Kruskal-Wallis test followed by post hoc Mann-Whitney U test. *∗∗* P < 0.01; *∗∗∗* P < 0.001. Data were expressed as the mean ± SD.

RA is always associated with pathologically increased osteoclastogenesis process. As moxibustion attenuated the pathological changes of RA, we then assessed the proteolytic activity of osteoclast to investigate whether moxibustion can inhibit the osteoclastogenesis process. We conducted histological staining to analysis the number of osteoclast. The obtained staining images determined a significant increase of osteoclast in RA rabbits (3.92 ± 0.25) when compared with control group (0.8 ± 0.07) (Figures 4(a), 4(b), and 4(d)), which were located in the damage area of bone trabeculae. However, moxibustion treatment to RA rabbits significantly decreased the number of osteoclast (1.74 ± 0.15) with respect to RA group (Figures 4(c) and 4(d)), which indicated that moxibustion inhibited the osteoclastogenesis process in RA.

### 3.3. Moxibustion Treatment Restores the Expression of RANKL/OGP in RA

**Figure 5:**
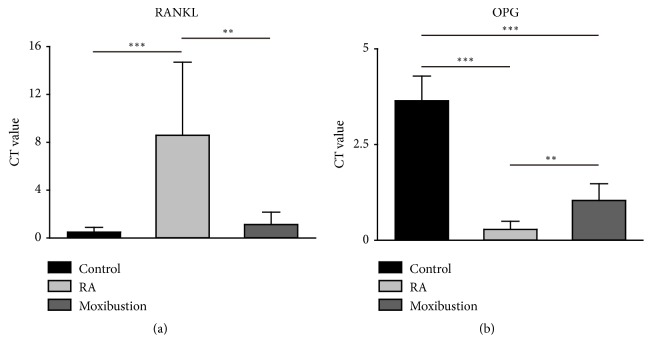
**Moxibustion of Zusanli and Shenshu bidirectionally regulates the expression of RANKL and OPG mRNA in RA**. (a) Moxibustion decreases the level of RANKL mRNA. (b) Moxibustion increases the level of OPG mRNA. n =10 for each group, the Kruskal-Wallis test followed by post hoc Mann-Whitney U test. *∗∗* P < 0.01; *∗∗∗* P < 0.001. Data were expressed as the mean ± SD.

As histological staining images provided the evidence of decreased osteoclast's proteolytic activity upon moxibustion treatment in RA rabbits, we next performed real-time PCR to analyze the osteoclastogenic factors OPG and RANKL at mRNA level. The quantitative data showed a significant fold change increase in RANKL (~ 8.5-fold) (Figure 5(a)), whereas OPG mRNA level was suppressed (~ 0.28-fold) (Figure 5(b)) in comparison with control rabbits. In contrast, moxibustion treatment to RA rabbits showed downregulated expression of RANKL (~ 1.11-fold) (Figure 5(a)) and abundant OPG (~ 1.03-fold) (Figure 5(b)) with respect to RA rabbits. Taken together, these results indicated that moxibustion treatment inhibited the osteoclast's proteolytic activity through regulation of RANKL/OPG pathway in RA.

## 4. Discussion

RA is a chronic autoimmune disease that causes inflammation of the joints. The incidence of RA increases with age and most RA patients suffer reduced physical function and progressive disability. Due to its obscure etiology and systemic complications, RA is treated with an interdisciplinary approach that consists of pharmacotherapy, biological therapy, physical therapy, and patient education [21]. Disease modifying antirheumatic drugs (DMARDs) and nonsteroidal anti-inflammatory drugs (NSAIDs) are important components of the pharmacotherapeutic approach. Although early introduction of DMARDs and NSAIDs provides great health benefits, these drugs have shown some serious limitations; for example, administration of NASIDs dose has benefit on bone homeostasis but has not been found to slow disease progression [22, 23]. Biological agents are alternative treatment options with documented effect for reducing inflammation of joint destruction; however, it entails a risk of resistance development and the high cost [24]. Therefore, RA patients sustaining with these conventional drugs are increasingly using other substitutes like complementary and alternative medicine (CAM) modalities for treatment [25]. Among the CAM treatments, natural plant products comprise one of the most widely used CAM for inflammatory and immune diseases. Moxibustion is manipulated with burning moxa (dried leaves from mugwort,* Artemisia vulgaris* from traditional Chinese medicine) to specific acupoints of body. The mechanism of moxibustion is mainly mediated by combined effects of the burned agent in the moxa and the special role of the acupoints. The thermal effects, radiation effects, and combustion products of moxa penetrate through and into the acupoints, which functions in dredging the meridians, harmonizing the Qi-blood and regulating the organs [26]. In the present study, we investigated the antiarthritic effects of moxibustion on a RA rabbit model.

Articular bone loss is the most prominent characteristic of RA, beginning early and persisting with disease progression. Articular cartilage erosion is particularly important clinically, as it is strongly related to function disability [27]. The delicate matched balance between bone resorption and bone formation seen in physiological states is disturbed in RA. Articular cartilage erosion results from excessive bone resorption and markedly diminished bone formation. Current therapeutic approaches have the potential to slow or even halt the progression of articular cartilage erosion; however, repair of the already existing cartilage erosion is observed infrequently (~ 10%) [28]. In the present study, we found that moxibustion of Zusanli (ST36) and Shenshu (BL23) acupoints alleviated the pathological progression of RA. Articular cartilage surface damage, tidemark destruction, and subchondral bone trabeculae loss were improved in moxibustion group compared with RA group. Together with previous study demonstrating that moxibustion alleviated the knee swelling in RA rabbits [29], our results suggested that moxibustion has a potent capability to prevent diseases progression and repair the established cartilage erosion, at least in part. However, whether moxibustion can improve the movement ability of RA rabbit model needs to be further investigated.

The balance between bone resorption and bone formation in physiological conditions is maintained by the coordinated activity of osteoblasts and osteoclasts. A cascade of inflammatory activity stimulates osteoclasts differentiation and maturation and impairs osteoblast differentiation and function, resulting in the net bone loss and bone erosion in RA [5]. The inflammatory cytokines, such as TNF-*α*, IL-6, and IL1-*β*, are critical nonenzymatic mediators involved in the bone resorption process. These inflammatory mediators activate the osteoclast directly or synergies with RANKL for promotion of osteoclastogenesis [18]. Previous studies using 3 weeks of direct moxibustion of Zusanli (ST36) and Shenshu (BL23) decreased the level of TNF-*α* and IL1-*β* in the synovial fluid of articular cavities in RA rabbit model [29], indicating a role of moxibustion in downregulation of inflammatory cytokines. However, whether moxibustion has treatment-duration dependent effects on inflammatory cytokines is urgently needed. Of note, stress is related to inflammatory response and moxibustion itself could be a stressor under some circumstances, such as inaccurate manipulation or improper treatment of animals [30]. In the present study, we found that rabbits were relaxed during moxibustion treatment, indicating rabbits were not in a stressful condition. Moreover, moxibustion of Zusanli (ST36) has antistress effects by decreasing the hypothalamic corticotropin-releasing hormone level and downregulating the activities of hypothalamic-pituitary-adrenal axis [31], indicating the observed phenomena in the present study were not disturbed with stress. In this study, increased number of osteoclast is observed in the articular cartilage in RA group, which is responsible for the cartilage degradation and bone erosion. Our experimental study proved that moxibustion treatment inhibited the increase of osteoclast, indicating a possible role of moxibustion in restoration of the bone metabolism homeostasis through its anti-inflammatory effects. However, moxibustion affects which period (migration, fusion, activation, or survival) of osteoclastogenesis and how moxibustion exerts these effects needs to be further examined.

RANKL plays an essential role in the orchestration of the osteoclast maturation, activation, migration, and survival [6]. In this study, increased RANKL was observed in RA established rabbits. Previous studies indicated that RANKL binds to RANK expressed in osteoclast precursor cells to promote osteoclast differentiation and function [32]. In addition, mature osteoblasts and other cells secrete OPG to inhibit RANKL activity to achieve bone homeostasis through preventing osteoclastogenesis and subsequent bone resorption [11]. The present results suggested that moxibustion decreased RANKL mRNA expression and conversely increased OPG mRNA expression in the current rabbit model of RA. The regulation of OPG/RANKL activity alongside with suppression of cartilage degradation in the current study indicated that the effect of moxibustion on RA may be associated with the OPG/RANKL pathway. In conclusion, the present results indicated that moxibustion had an antiarthritic effect by regulating OPG/RANKL pathways. However, the specific mechanisms by which moxibustion affects RA remain to be elucidated.

## 5. Conclusions

The present study provided in vivo experimental based data that moxibustion of Zusanli (ST36) and Shenshu (BL23) acupoints alleviated the cartilage degradation and bone destruction in a rabbit model RA. Furthermore, RT-PCR results indicated that the effects of moxibustion on RA may be associated with regulation of OPG/RANKL signaling.

## Data Availability

The data used to support the findings of this study are available from the corresponding author upon request.
